# RNF219 RING Finger Domain Mutants Drive Phase Separation to Encapsulate CCR4‐NOT and Promote Cell Proliferation

**DOI:** 10.1111/cpr.70072

**Published:** 2025-06-11

**Authors:** Chen Chen, Chenghao Guo, Ke Fang, Chengqi Lin, Zhuojuan Luo

**Affiliations:** ^1^ Key Laboratory of Developmental Genes and Human Disease School of Life Science and Technology, Southeast University Nanjing China; ^2^ Co‐Innovation Center of Neuroregeneration Nantong University Nantong China; ^3^ Shenzhen Research Institute Southeast University Shenzhen China

**Keywords:** CCR4‐NOT, LLPS, mutants, RNF219

## Abstract

RING finger protein 219 (RNF219) is a co‐factor for the CCR4‐NOT deadenylase complex in mammals. Here, we found that mutations within the C3HC4 scaffold of the RING finger domain in RNF219 are capable of forming condensates via liquid–liquid phase separation (LLPS), though the wild‐type RING finger domain intrinsically suppresses LLPS. We further demonstrated that the adjacent coiled‐coil 1 (CC1) domain promotes the potential of RNF219 to form condensates. Moreover, the mutant RNF219 condensates are able to encapsulate the CCR4‐NOT complex, inhibiting the RNA deadenylation activity of CCR4‐NOT. Additionally, we observed that RNF219 mutations could promote cell proliferation. These findings suggest a pathogenic mechanism whereby RNF219 mutations could induce CCR4‐NOT condensate formation, inhibit deadenylation‐dependent mRNA decay and drive cell proliferation.

## Graphical abstract

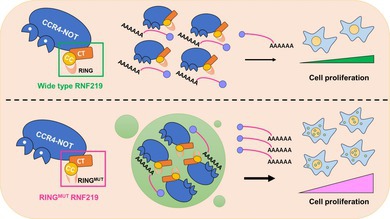

## 1 Introduction

RNA decay is a crucial step in post‐transcriptional regulation. In eukaryotic cells, the CCR4‐NOT complex plays a central role in this process [1]. The CCR4‐NOT complex is a multifunctional assembly composed of multiple subunits. Among its components, CNOT1 serves as a structural scaffold, enabling the recruitment and interaction of other subunits [2]. CNOT2, CNOT3 and CNOT9 associate with various RNA‐binding proteins (RBPs), thereby targeting the complex to RNA substrates [3, 4, 5, 6]. The CCR4 (CNOT6/CNOT6L) and CAF1 (CNOT7/CNOT8) subunits within the complex cooperatively mediate deadenylation activity, essential for mRNA turnover and stability regulation [7, 8, 9]. Notably, CNOT6 and CNOT7 exhibit distinct roles: CNOT7 processes free poly(A) tails unbound by PABP, whereas CNOT6 acts on PABP‐bound poly(A) tails [10, 11]. Furthermore, multiple subunits of the CCR4‐NOT complex coordinate through intrinsic regulatory mechanisms to collectively regulate poly(A) tail shortening [12].

Previously, we and others have identified RNF219 as a co‐factor of the CCR4‐NOT complex [13, 14, 15]. RNF219 interacts with the CCR4‐NOT complex through CNOT9 [14]. Functionally, RNF219 plays a crucial role in the neural differentiation of embryonic stem cells by enhancing mRNA stability [13]. Beyond this role, RNF219 promotes DNA replication by ubiquitinating the origin recognition complex (ORC) [16] and drives bone metastasis in hepatocellular carcinoma through activation of the Hippo and Wnt signalling pathways via the α‐Catenin‐LGALS3 axis [17]. Despite these findings, the molecular functions of RNF219 remain poorly understood, particularly with regard to its interactions with the CCR4‐NOT complex.

Liquid–liquid phase separation (LLPS) is a fundamental biological phenomenon that underpins diverse cellular processes [18, 19, 20]. The CCR4‐NOT complex is implicated in LLPS‐related phenomena; for instance, CNOT1 suppresses processing body (PB) assembly by destabilising Dhh1 condensates [21], whereas CNOT7 integrates into FMRP‐CAPRIN1‐RNA condensates, with its deadenylation activity modulated by FMRP and CAPRIN1 phosphorylation states [22]. Dysregulated phase separation has been implicated in the pathogenesis of various diseases [23, 24, 25, 26]. Here, we investigated RNF219 mutants in various patients with cancer and found that mutations within the C3HC4 scaffold of the RING finger domain are capable of driving RNF219 to undergo LLPS and form condensates. We further demonstrated that the CC1 domain of RNF219 is essential for condensate formation. Additionally, the mutant RNF219 condensates could encapsulate the CCR4‐NOT complex, suppressing its mRNA deadenylation activity and promoting tumour cell proliferation. This study provides valuable insights for understanding the role of RNF219 mutants in cancers.

## 2 Results

### 2.1 RNF219 Mutations in Patients With Cancer

To investigate the prevalence of RNF219 mutations in somatic cells of patients with tumour, we conducted a statistical analysis of mutation data from 31 projects available in The Cancer Genome Atlas (TCGA). A total of 172 cases involving 193 mutations were analysed, revealing that RNF219 mutations occurred in 6.25% of patients in TCGA–uterine corpus endometrial carcinoma (TCGA‐UCEC), 3.83% of patients in TCGA–skin cutaneous melanoma (TCGA‐SKCM) and 3.27% of patients in TCGA–colon adenocarcinoma (Figure S1B).

**FIGURE 1:**
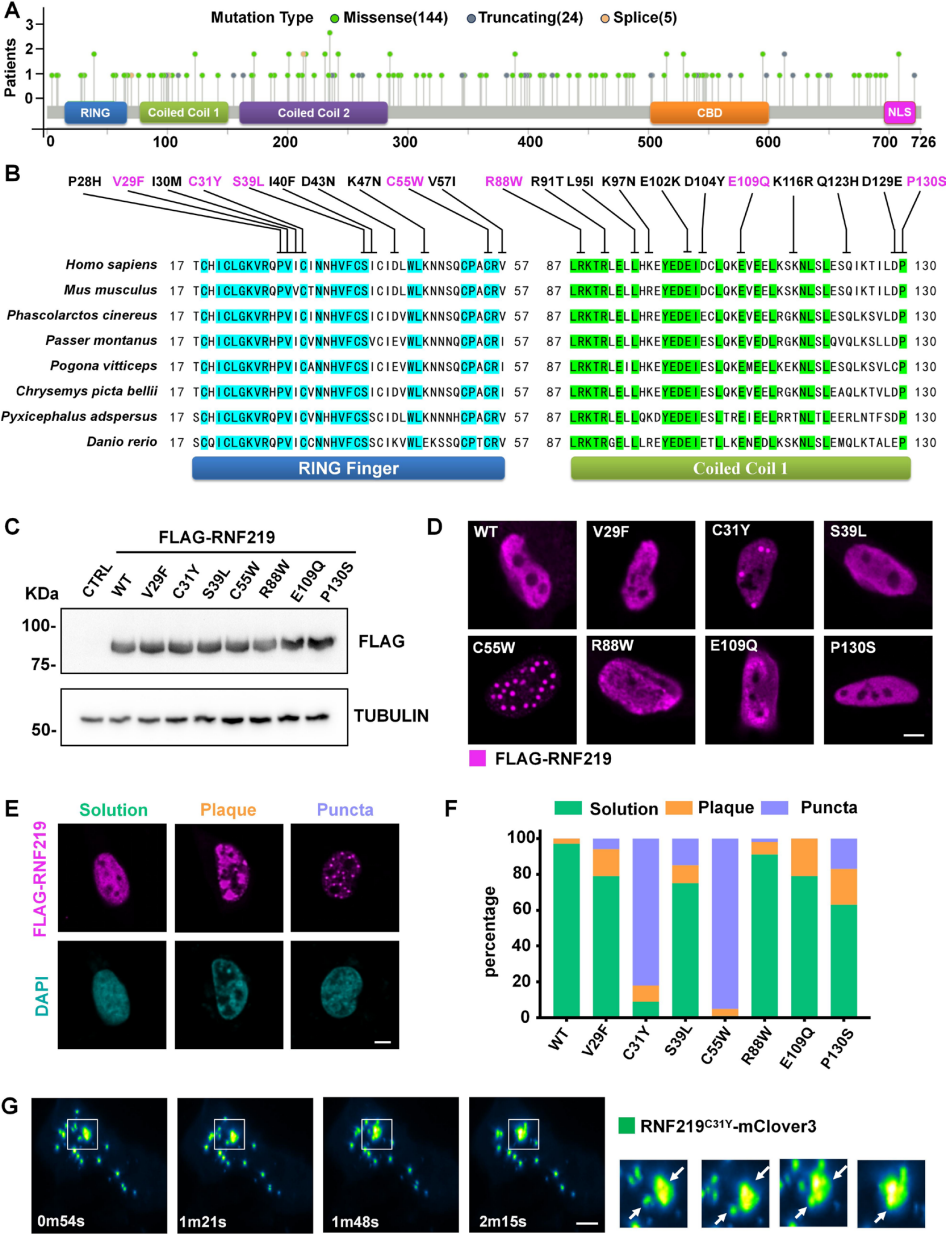
RNF219 mutants, RNF219^C31Y^ and RNF219^C55W^, formed condensates in cells. (A) *RNF219* cancer mutation plot. The green dots indicate the missense mutation, the grey dots indicate truncating, and the yellow dots indicate splice. (B) The species conservation analysis of the RNF219 RING and CC1 domains. Magenta labels mark the seven missense mutations identified in this study. (C) Western blot showing the protein levels of transfected FLAG‐tagged RNF219 and mutants in HEK293T cells. FLAG‐tagged RNF219 mutant proteins detected via anti‐FLAG antibody; TUBULIN was used as a loading control. (D) Representative confocal images of HeLa cells transfected with recombinant FLAG‐tagged RNF219 and mutants (V29F, C31Y, S39L, C55W, R88W, E109Q, P130S). Immunostained with anti‐FLAG antibody. Scale bar, 5 μm. (E) According to RNF219^WT^ and RNF219^Mut^ the different distribution states of proteins in the nucleus of HeLa, representative images are divided into three categories: solution, plaque and puncta. Immunostained with anti‐FLAG antibody; DNA was counterstained using DAPI. Scale bar, 5 μm. (F) Quantification results of (D). Green was solution, yellow was plaque, and blue was puncta (*n* ≥ 50). (G) Time‐lapse fluorescence images of a HeLa cell expressing RNF219^C31Y^‐mClover3 subjected to illuminate every 27 s for the times indicated. The two RNF219^C31Y‐^mClover3 droplets underwent spontaneous fusion as indicated by arrows. Scale bar, 5 μm.

Mutation profiling revealed that RNF219 mutations are distributed across the entire protein without distinct clustering (Figure 1A). Sequence conservation analysis highlighted high conservation of the RING and CC1 domains across vertebrates (Figure S1C). Focusing on conserved residues within these domains, we selected seven missense mutations (V29F, C31Y, S39L, C55W, R88W, E109Q and P130S) for functional investigation (Figure 1B). To validate expression levels, we compared endogenous RNF219 with FLAG‐tagged overexpression variants in HEK293T and HeLa cells. Overexpressed proteins exhibited markedly higher levels than endogenous counterparts (Figure S2A). FLAG‐tagged RNF219 mutants expressed in HEK293T cells showed protein levels comparable to wild‐type (WT) RNF219 (Figure 1C).

A prior study by Poetz et al. [14] in 2021 suggested that mutations in conserved cysteine residues of the RING finger C3HC4 scaffold enhance protein stability. To evaluate whether single‐point mutations in this motif affect RNF219 stability, we performed cycloheximide (CHX) chase assays in HEK293T cells. Western blot (WB) quantification revealed similar half‐lives for WT (3.0 h), V29F (3.2 h), C31Y (3.2 h) and C55W (3.5 h), with no statistically significant differences (Figure S2B).

### 2.2 RNF219^C31Y^ and RNF219^C55W^ Could Form Condensates in Cells

To determine whether RNF219 mutations affect cellular localization of RNF219, we expressed FLAG‐tagged RNF219 mutants (RNF219^V29F^, RNF219^C31Y^, RNF219^S39L^, RNF219^C55W^, RNF219^R88W^, RNF219^E109Q^ and RNF219^P130S^) and analysed their distribution via immunofluorescence in HeLa cells. The results showed that the subcellular distribution of these RNF219 mutants differed (Figure 1D). Based on their observed condensate states, we categorised them into three types for statistical analysis: solution (dispersed), plaque (patch‐like) and puncta (droplet‐like) (Figure 1E). Interestingly, although RNF219^WT^ predominantly remained in the solution state, these mutations promoted a transition from the solution state to the plaque or puncta states. Notably, C31Y and C55W mutations substantially enhanced the puncta formation capability of RNF219 (Figure 1F).

LLPS has emerged as a fundamental mechanism regulating various biological processes. Although C55W also formed condensates in cells (Figure 1F), C31Y exhibited higher consistency across replicates and was prioritised for further analysis. We selected the RING domain mutants V29F (non‐puncta forming) and C31Y (puncta forming) as representative variants. We expressed RNF219^WT^, RNF219^V29F^ and RNF219^C31Y^ fused to a C‐terminal mClover3 tag in HeLa cells. Live‐cell imaging revealed distinct condensates formed by RNF219^C31Y^‐mClover3 (Figure S2C,D). Similar phenotypes were observed with mCherry‐tagged variants (Figure S2E,F). Additionally, the expression of RNF219^C31Y^‐mClover3 in other cell lines also confirmed the presence of puncta (Figure S2G,H). Furthermore, live‐cell imaging revealed fusion events between two RNF219^C31Y^‐mClover3 droplets (Figure 1G). These findings indicate that RNF219^C31Y^ puncta exhibit dynamic liquid‐like behaviour.

### 2.3 Mutations in the RING Finger C3HC4 Scaffold Drive RNF219 Condensates Formation

**FIGURE 2:**
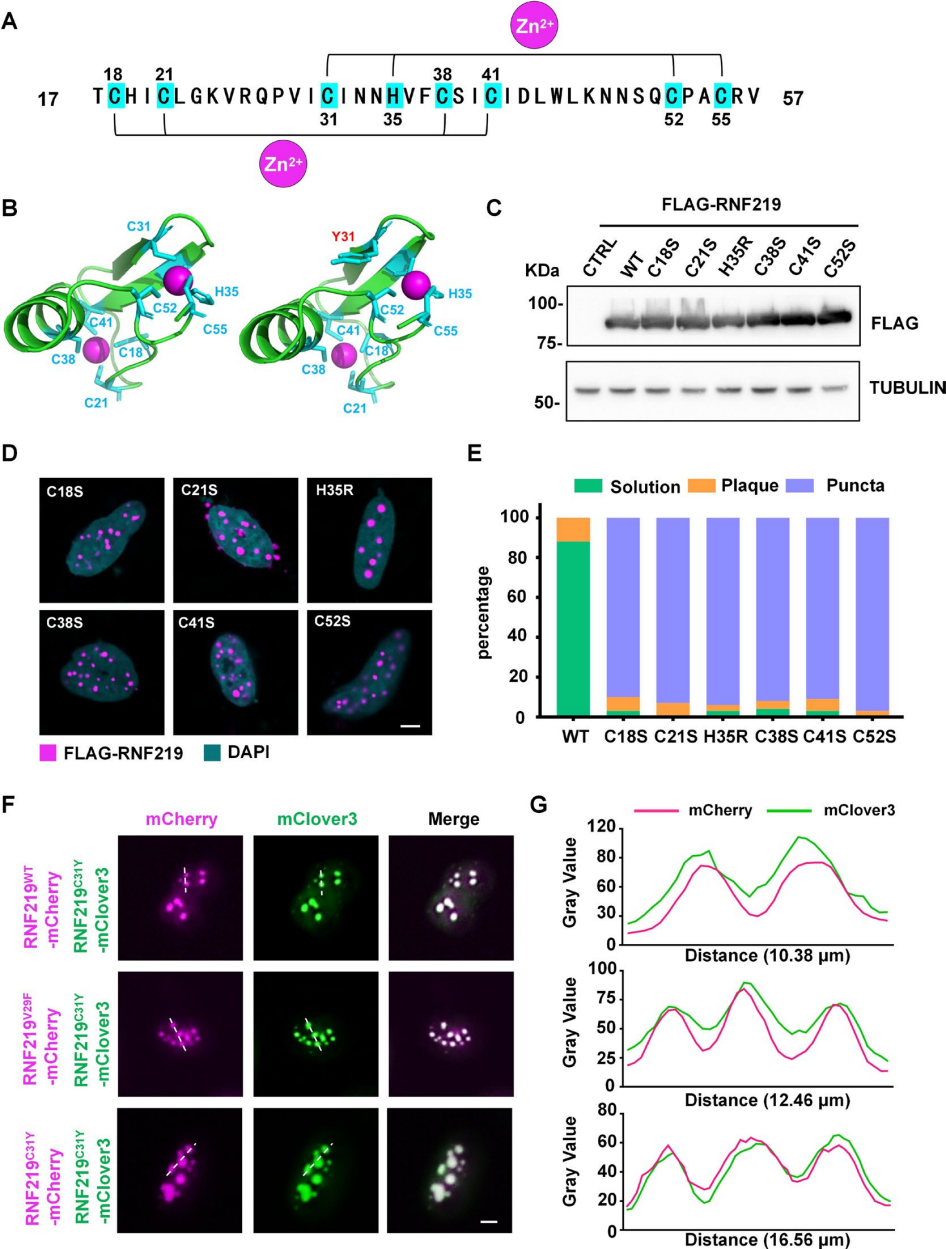
Mutations in the RING finger C3HC4 scaffold drove RNF219 condensates formation. (A) The alignment of the RING finger domain of RNF219. Blue labels marked C3HC4 amino acids interact with Zn^2+^. (B) AlphaFold3 (https://alphafoldserver.com/) predicted structure and sequence analysis of the RNF219 RING finger domain (18‐57aa) interact with Zn^2+^, WT (left) and C31Y (right). Blue mark the C3HC4 amino acids and red mark C31Y mutation. The analysis and image export using PyMOL. (C) Western blot showing the protein levels of transfected FLAG‐tagged RNF219 and mutants (C18S, C21S, H35R, C38S, C41S, C52S) in HEK293T cells. FLAG‐tagged RNF219 mutant proteins detected via anti‐FLAG antibody, TUBULIN was used as a loading control. (D) Representative confocal images of HeLa cells transfected with recombinant FLAG‐tagged RNF219 and mutants (C18S, C21S, H35R, C38S, C41S, C52S). Immunostained with anti‐FLAG antibody. DNA was counterstained using DAPI. Scale bar, 5 μm. (E) Quantification result of (D) was shown. Green was solution, yellow was plaque, and blue was puncta (*n* ≥ 25). (F) Live cell imaging of HeLa cells co‐expressed RNF219^C31Y^‐mClover3 and RNF219^WT^‐mCherry, RNF219^V29F^‐mCherry or RNF219^C31Y^‐mCherry. Scale bar, 5 μm. (G) Line intensity profiling of representative sections of cells in (F) is indicated by white dashed lines.

The RING finger domain of RNF219 contains a C3HC4 scaffold (Figure 2A), where conserved cysteine and histidine residues are embedded within the domain's core, maintaining its structural integrity by coordinating two Zn^2+^ [27]. Using AlphaFold3, we predicted structural changes in the RING domain caused by the C31Y mutation. The mutation disrupted the domain pocket, impairing Zn^2+^ binding (Figure 2B). Given that both C31Y and C55W mutations occur at cysteine residues within the C3HC4 scaffold of the RING finger domain, we hypothesised that one of these amino acids in C3HC4 could promote the formation of RNF219 condensates. To test this, we generated FLAG‐tagged point mutants (C18S, C21S, H35R, C38S, C41S and C52S) within the C3HC4 scaffold, expressed in HEK293T cells (Figure 2C) and analysed their subcellular distribution in HeLa cells via immunofluorescence. Strikingly, all mutants formed condensates (Figure 2D,E), indicating that any mutation in the C3HC4 motif induces RNF219 LLPS. Among tested mutants, C31Y exhibited the most robust and reproducible phase‐separation phenotype. We thus selected this mutant for detailed functional characterisation.

To assess the dominant effect of C31Y condensates, we co‐expressed mClover3‐tagged RNF219^C31Y^ with mCherry‐tagged RNF219^WT^, RNF219^V29F^, or RNF219^C31Y^ in HeLa cells. Live‐cell imaging and line intensity analysis revealed efficient incorporation of WT and V29F variants into C31Y condensates (Figure 2F,G), despite their inability to form puncta when expressed alone (Figure S2E,F). This demonstrates that C31Y condensates could sequester non‐phase‐separating RNF219 variants.

### 2.4 The CC1 Domain Induced LLPS Formation, and RING Finger Domain Suppressed It

**FIGURE 3:**
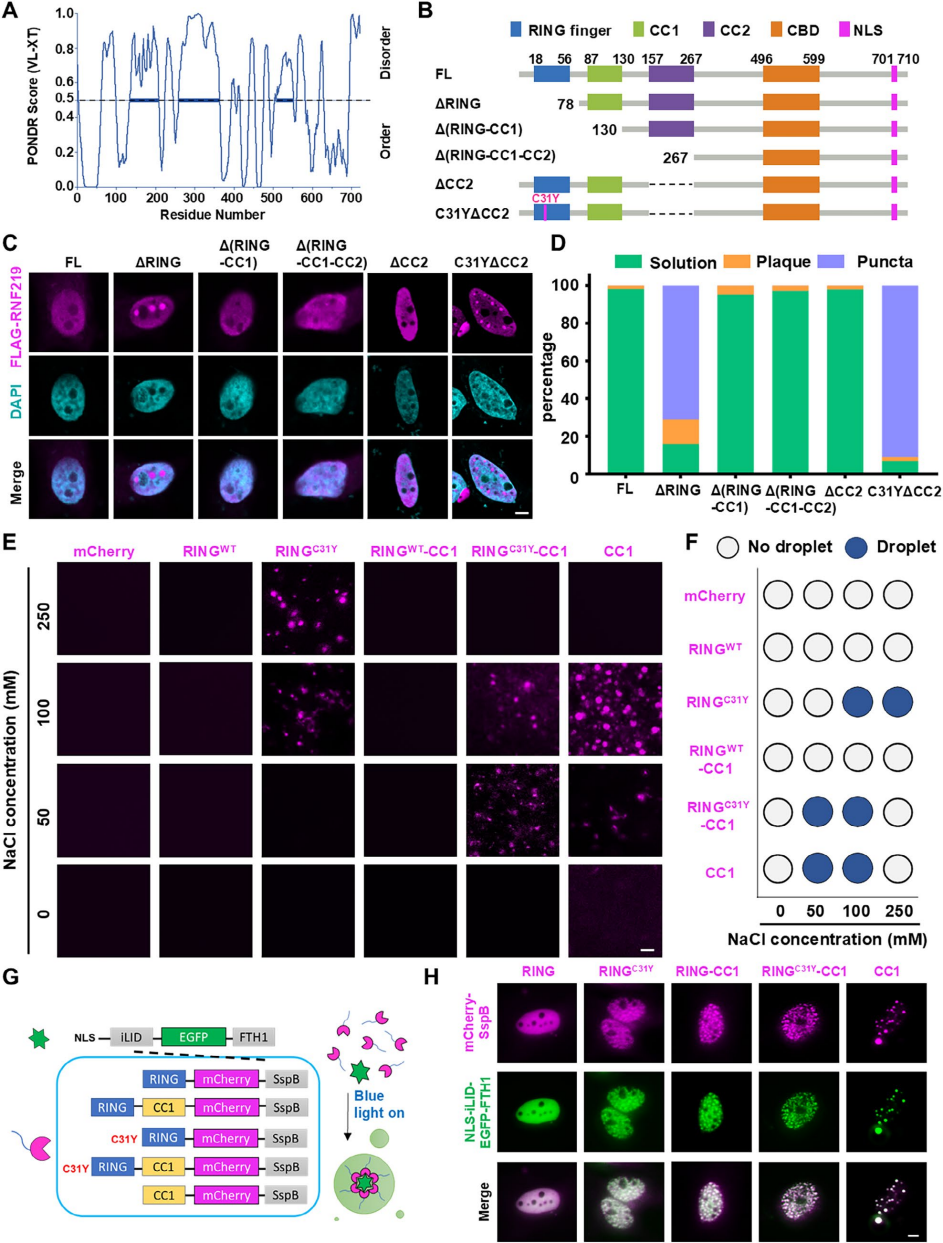
The CC1 domain induced LLPS formation and the RING finger domain suppressed it. (A) Graph plots showing the intrinsic disorder regions across the entire length of RNF219 calculated by PONDR VS‐XT algorithm. The intrinsic scores are shown on the *y*‐axis, and amino acid positions are shown on the *x*‐axis. The scores are assigned between 0 and 1, and the score above 0.5 indicates disorder regions. (B) Schematic diagram of the full‐length (FL) and truncation variants, including ΔRING, Δ(RING‐CC1), Δ(RING‐CC1‐CC2), ΔCC2 and C31YΔCC2 of the RNF219 protein. (C) Representative confocal images of HeLa cells transfected with recombinant FLAG‐tagged RNF219 FL and truncations. Immunostained with anti‐FLAG antibody, DNA was counterstained using DAPI. Scale bar, 5 μm. (D) Quantification result of (C) was shown. Green was solution, yellow was plaque, and blue was puncta (*n* ≥ 25). (E) Fluorescence microscopy images showing phase‐separated droplets formed with mCherry, RING^WT^‐mCherry, RING^C31Y^‐mCherry, RING^WT^‐CC1‐mCherry, RING^C31Y^‐CC1‐mCherry, and CC1‐mCherry proteins with 10 μM concentration in 20 mM Tris (pH 8.0), 10% (w/v) PEG8000 in different sodium chloride containing buffer. Scale bar, 5 μm. (F) Phase diagrams of (E), white dots, no droplet; blue dots, droplet. (G) Schematic of the light‐activated intracellular droplet condensation system. (H) Representative images of live HeLa cells expressing RING^WT^, RING^C31Y^, CC1, RING^WT^‐CC1 and RING^C31Y^‐CC1 fusion proteins. Cells were stimulated with 488 nm blue light for 2 min. Scale bar, 5 μm.

We next sought to investigate the molecular mechanism underlying the formation of condensates by RNF219^Mut^. An analysis of the intrinsically disordered regions (IDRs) in the RNF219 protein revealed that the CC1 and CC2 domains contain IDRs, whereas the RING finger domain is highly ordered (Figure 3A). To further investigate, we expressed a series of truncated FLAG‐RNF219 constructs in HeLa cells (Figure 3B and Figure S3A). The results showed that RNF219 lacking the RING finger domain (ΔRING) was capable of forming condensates. However, truncation of both the RING finger and CC1 domains, Δ(RING‐CC1) abolished condensate formation (Figure 3C,D). These findings indicate that the CC1 domain could be essential for driving RNF219 condensate formation, particularly in the absence of the RING finger domain.

Given the IDR properties of CC2, we tested its role in RNF219^C31Y^‐mediated LLPS by generating ΔCC2 and C31YΔCC2 mutants (Figure 3B and Figure S3B). Immunofluorescence revealed that ΔCC2 failed to form condensates, whereas C31YΔCC2 retained this capacity. Combined with previous observations that ΔRING promotes condensates but Δ(RING‐CC1) (retaining CC2) does not (Figure 3C,D), these findings collectively demonstrate that CC2 is dispensable for LLPS in RNF219 mutants.

To assess phase separation potential in vitro, we purified mCherry‐fused recombinant proteins: RING^WT^, RING^C31Y^, RING^WT^‐CC1, RING^C31Y^‐CC1 and CC1 (Figure S3C). Under 10‐μM protein and 10% PEG8000 conditions, salt concentration gradients were tested. RING^C31Y^‐CC1 and CC1 formed condensates at 50–100 mM NaCl, whereas RING^C31Y^ required higher salt concentrations (100–250 mM). Neither RING^WT^ nor RING^WT^‐CC1 exhibited condensation at any tested concentration (Figure 3E,F), confirming that the WT RING domain suppresses CC1‐driven LLPS.

Furthermore, we used the light‐activated intracellular droplet condensation system [28] to further confirm these findings (Figure 3G). We observed that RING^C31Y^, RING^WT^‐CC1 and RING^C31Y^‐CC1, and CC1 formed condensates within 2 min of blue light activation. Notably, the RING finger domain alone failed to form condensates even after 5 min of blue light activation (Figure 3H). These findings establish that RNF219 phase separation capacity is governed by an antagonistic interplay between its structural domains: The CC1 domain acts as an LLPS driver, whereas the RING finger domain exerts autoinhibitory control over condensate formation.

### 2.5 CCR4‐NOT Complex Is Encapsulated in RNF219^Mut^ Condensates

**FIGURE 4:**
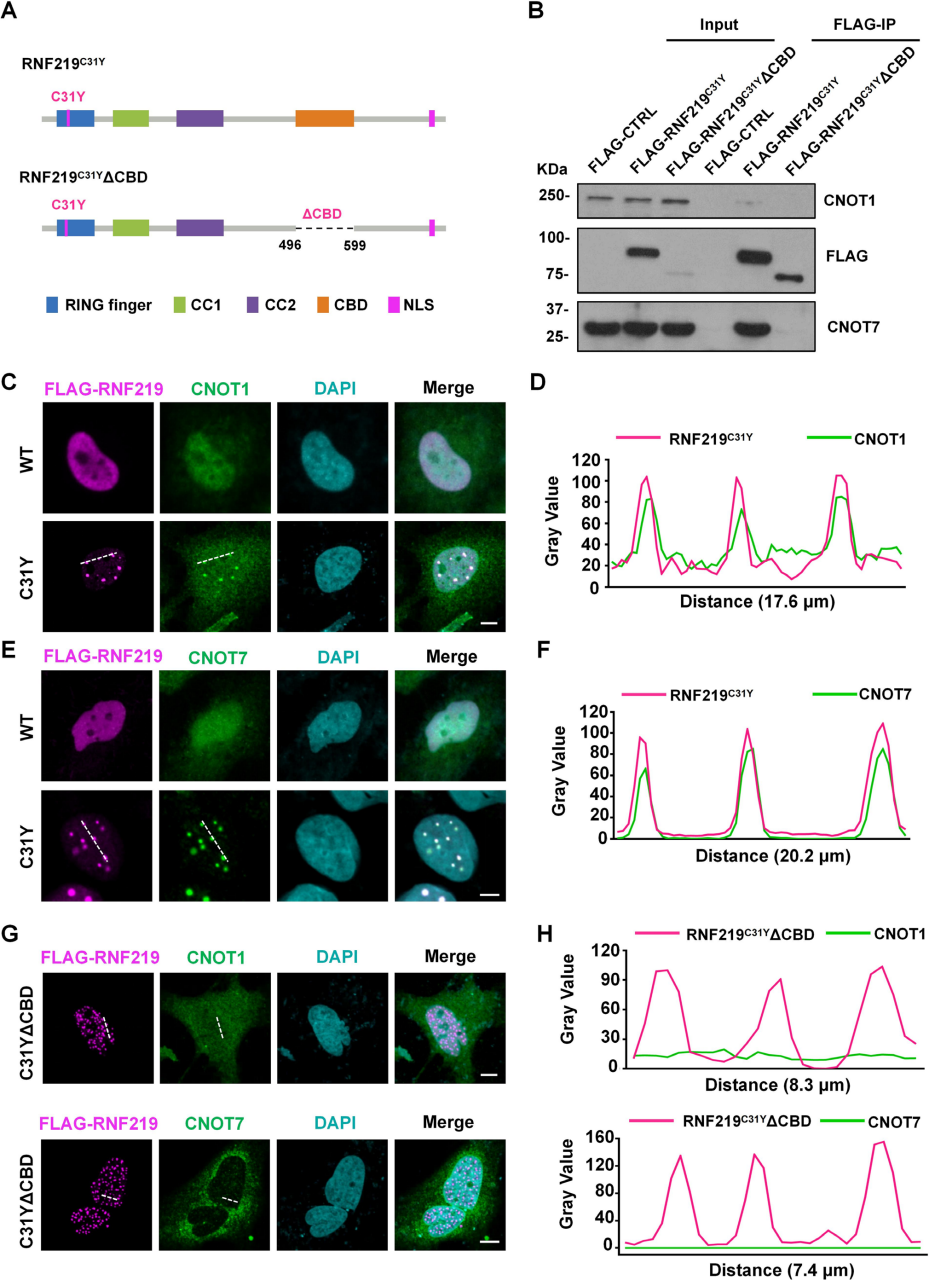
CCR4‐NOT complex was encapsulated in RNF219^Mut^ condensates. (A) Schematic diagram of the RNF219^C31Y^ and RNF219^C31Y^ΔCBD. RNF219 binds to CNOT9 via CBD. (B) Co‐immunoprecipitation assay demonstrating the interaction of FLAG‐tagged RNF219^C31Y^ or RNF219^C31Y^ΔCBD with CNOT1 and CNOT7. FLAG‐tagged RNF219 constructs detected via anti‐FLAG antibody. (C) Representative confocal images of HeLa cells transfected with recombinant FLAG‐tagged RNF219^WT^, RNF219^C31Y^. Immunostained with anti‐FLAG and anti‐CNOT1 antibodies, DNA was counterstained using DAPI. Scale bar, 5 μm. (D) Line intensity profiling of representative sections of cells in (C) is indicated by white dashed lines. (E) Representative confocal images of HeLa cells transfected with recombinant FLAG‐tagged RNF219^WT^, RNF219^C31Y^. Immunostained with anti‐FLAG and anti‐CNOT7 antibodies, DNA was counterstained using DAPI. Scale bar, 5 μm. (F) Line intensity profiling of representative sections of cells in (E) is indicated by white dashed lines. (G) Representative confocal images of HeLa cells transfected with recombinant FLAG‐tagged RNF219^C31Y^ΔCBD. Immunostained with anti‐FLAG and anti‐CNOT1 (top) or anti‐CNOT7 (bottom) antibodies, DNA was counterstained using DAPI. Scale bar, 5 μm. (H) Line intensity profiling of representative sections of cells in (G) is indicated by white dashed lines. CNOT1 (top) or CNOT7 (bottom).

RNF219 mediates its interaction with CNOT9 via a C‐terminal CCR4‐binding domain (CBD) stably binds to the CCR4‐NOT complex (Figure S1A). Co‐immunoprecipitation assays demonstrated that the C31Y mutation does not disrupt the interaction between RNF219 and the CCR4‐NOT complex (Figure 4B). To determine if CCR4‐NOT components localise to RNF219^Mut^ condensates, we performed co‐immunostaining and line intensity analysis. FLAG‐tagged RNF219^C31Y^ nuclear condensates co‐localised with endogenous CNOT1 and CNOT7, whereas RNF219^WT^ and CCR4‐NOT components exhibited diffuse distributions (Figure 4C–F). This differential co‐localisation pattern demonstrates that the C31Y mutation disrupts RING‐mediated autoinhibition, enabling CC1‐dependent co‐condensation with CCR4‐NOT core components.

To investigate whether the interaction between RNF219 and the CCR4‐NOT complex is necessary for RNF219^Mut^‐CCR4‐NOT condensate formation, we deleted the CBD domain from RNF219^C31Y^ (RNF219^C31Y^ΔCBD, Figure 4A). Immunoprecipitation analysis revealed that CBD deletion abolished CCR4‐NOT binding (Figure 4B). Despite the loss of the CBD, RNF219^C31Y^ΔCBD still formed condensates in cells. However, CNOT1 and CNOT7 did not colocalize with RNF219^C31Y^ΔCBD and instead exhibited a dispersed fluorescence pattern (Figure 4G,H). These results suggest that RNF219^Mut^‐CCR4‐NOT complex condensate formation is dependent on protein–protein interactions.

### 2.6 RNF219 Mutations Inhibit Deadenylation Activity of CCR4‐NOT In Vitro and Induce Cell Proliferation

**FIGURE 5:**
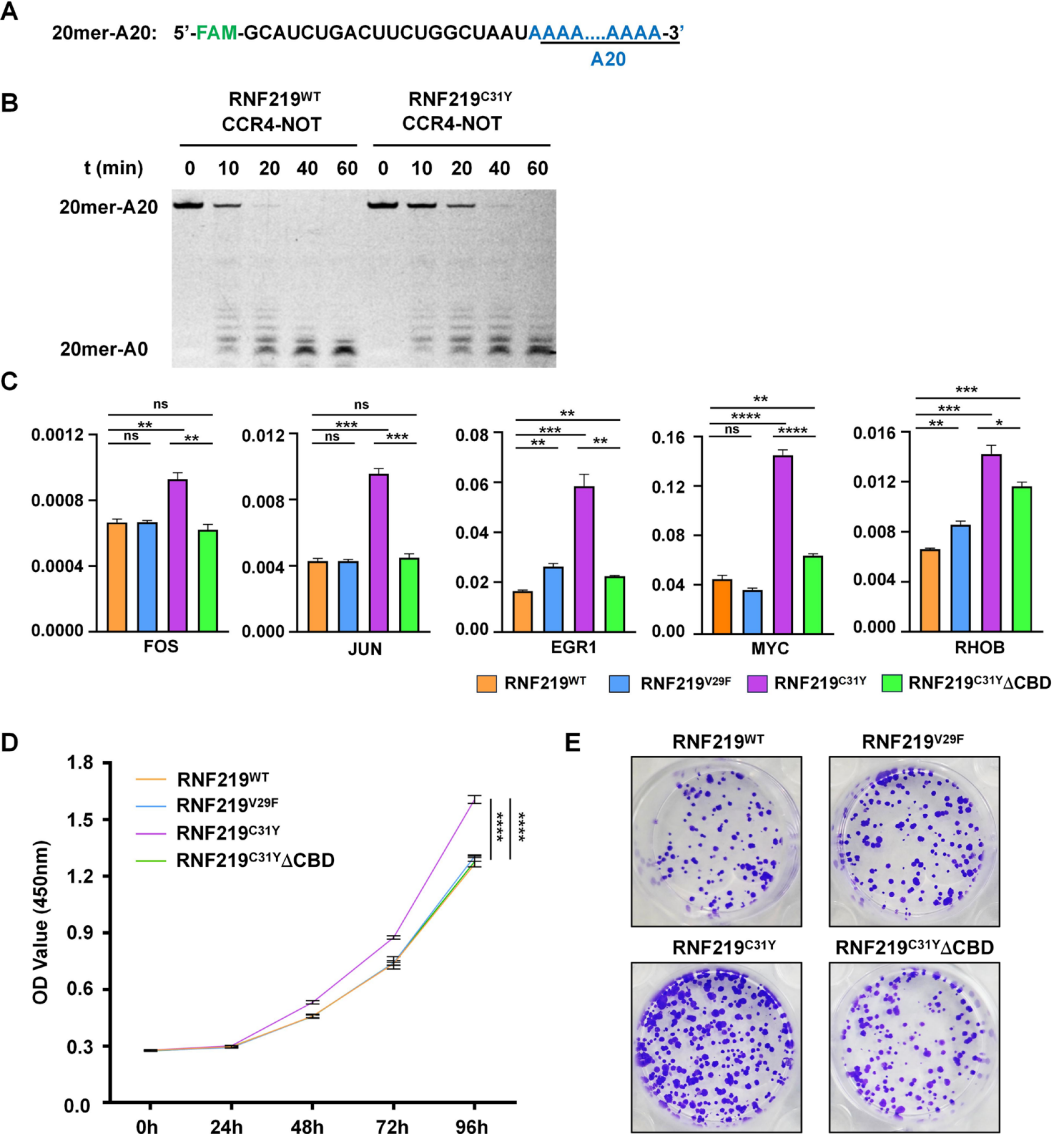
RNF219^Mut^‐CCR4‐NOT condensates promoted cell proliferation by inhibiting deadenylation. (A) RNA substrate used in the in vitro deadenylation assay. (B) In vitro RNA deadenylation assay of the RNF219‐CCR4‐NOT complex. Reactions were stopped at the indicated time points and analysed by electrophoresis using a 20% denaturing polyacrylamide gel. (C) RT‐qPCR analysis showing the relative expression levels of *FOS*, *JUN*, *EGR1*, *MYC* and *RHOB* in DLD‐1 cells expressing FLAG‐tagged RNF219^WT^, RNF219^V29F^, RNF219^C31Y^ or RNF219^C31Y^ΔCBD. Data are presented as means ± SEM. ns, not significant; *, *p* < 0.05; **, *p* < 0.01; ***, *p* < 0.001; ****, *p* < 0.0001. (D) Quantifying viable cell numbers in proliferation in DLD‐1 cells expressing FLAG‐tagged RNF219^WT^, RNF219^V29F^, RNF219^C31Y^ or RNF219^C31Y^ΔCBD. Data are presented as means ± SEM. ns, not significant; *, *p* < 0.05; **, *p* < 0.01; ***, *p* < 0.001; ****, *p* < 0.0001. (E) Crystal violet staining showing the clonogenic assay of DLD‐1 cells expressing FLAG‐tagged RNF219^WT^, RNF219^V29F^, RNF219^C31Y^ or RNF219^C31Y^ΔCBD.

We previously showed that the RNF219‐CCR4‐NOT complex exhibits deadenylation activity in vitro [13, 14]. To assess the impact of the C31Y mutation, we purified the RNF219^WT/C31Y^‐CCR4‐NOT complex (Figure S4A) and measured CCR4‐NOT‐mediated degradation of a 5′‐fluorescein‐labelled RNA substrate (20mer‐A20; Figure 5A). RNF219^C31Y^ significantly reduced deadenylation activity compared with WT (Figure 5B). Although this attenuation correlates with condensate formation, further studies are needed to establish causality.

To probe the pathological relevance of RNF219^Mut^, we stably expressed FLAG‐tagged RNF219^WT^, RNF219^V29F^, RNF219^C31Y^ and RNF219^C31Y^ΔCBD in the colorectal adenocarcinoma cell line DLD‐1 cells (Figure S4B,C). RNF219^C31Y^ΔCBD retained LLPS capacity but lost CCR4‐NOT interaction (Figure 4A,B,G,H). Based on prior links between RNF219 and mRNA stabilisation [14], we quantified mRNA levels of immediate‐early genes (*FOS*, *JUN*, *EGR1*), *MYC* and *RHOB* via RT‐qPCR. RNF219^C31Y^ upregulated these transcripts, whereas C31YΔCBD failed to do so (Figure 5C).

As these genes are associated with cell proliferation and cancer, we assessed the cell proliferation capacity of these DLD‐1 cells. Cell proliferation was quantified using the CCK‐8 assay kit. Meanwhile, we also used a clonogenicity assay to assess cell proliferation. Compared with RNF219^WT^ and RNF219^V29F^, RNF219^C31Y^ promoted cell proliferation. Moreover, RNF219^C31Y^ΔCBD also lost the ability to promote proliferation (Figure 5D,E). Taken together, these results suggest that the C31Y mutation in the RNF219 RING domain promotes cell proliferation.

## 3 Discussion

RNF219 has been implicated in regulating cell proliferation and embryonic stem cell differentiation [13, 16], with its overexpression promoting tumorigenesis and cancer progression [17, 29]. However, the functional consequences of RNF219 mutations remain poorly characterised. Here we found that mutations in the RING finger domain of RNF219 could lead to phase separation of RNF219. We further showed that RNF219 phase separation is mediated by its CC1 domain, which is intrinsically disordered. Interestingly, the RING finger domain located at the N‐terminus of RNF219 directly suppresses the ability of CC1 to induce phase separation. However, mutations in the C3HC4 motif of the RING finger domain reduce its inhibitory function. It is possible that the alteration in the balance between the RING and CC1 domains enables the mutated RNF219 to experience CC1‐induced phase separation. This finding suggests that the relationship between the RING and CC1 domains of RNF219 could be a key aspect in understanding the overall function and behaviour of RNF219.

**FIGURE 6:**
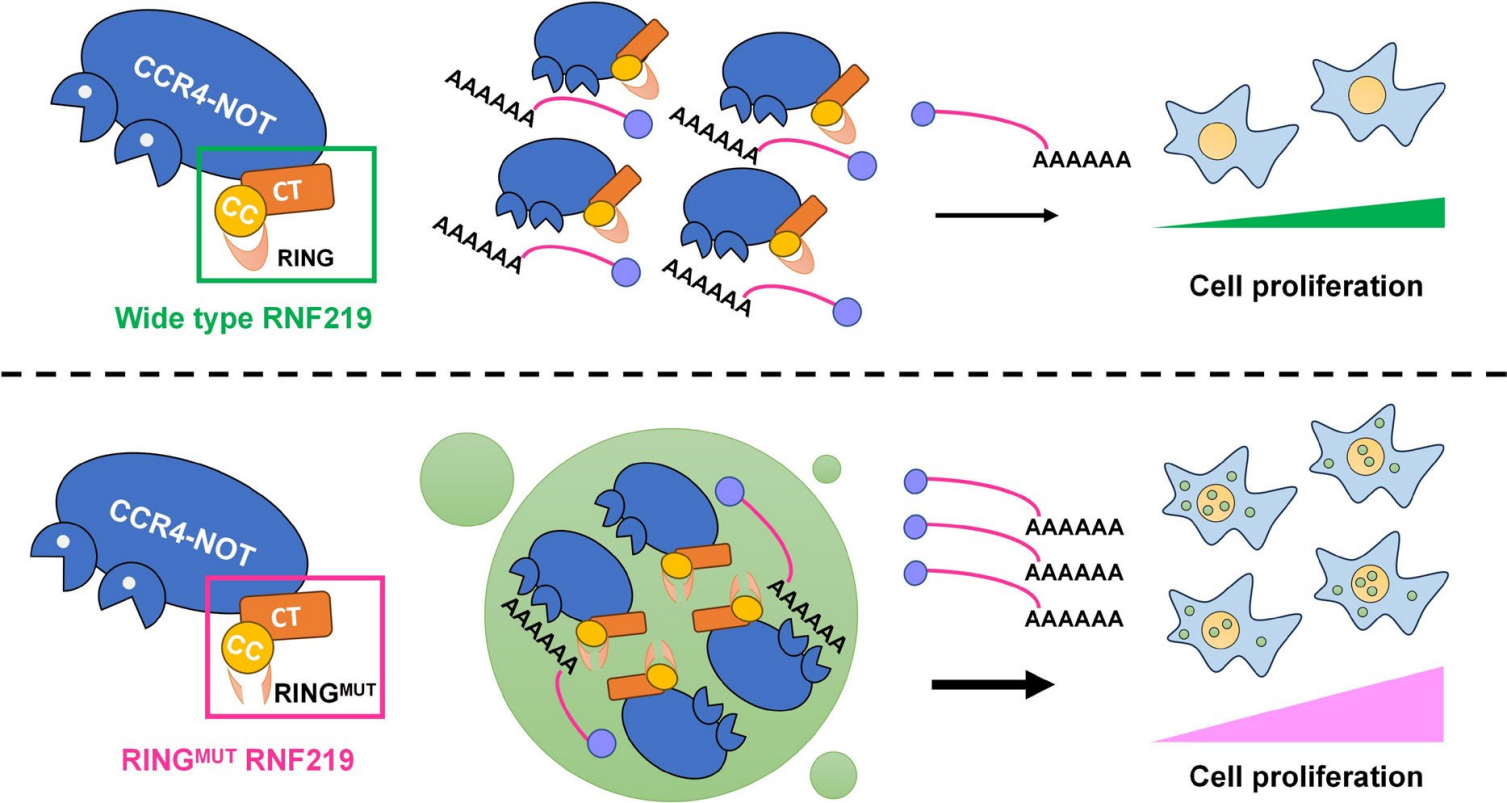
Schematic representation of RNF219Mut‐CCR4‐NOT condensates. Mutations in the RING finger domain (C3HC4) of RNF219 promote the formation of RNF219 condensates, which are able to encapsulate the CCR4‐NOT complex. These RNF219^Mut^‐CCR4‐NOT condensates reduce the deacetylation activity of the CCR4‐NOT complex and induce cell proliferation.

The CCR4‐NOT complex plays a central role in RNA decay, a crucial step in post‐transcriptional regulation in eukaryotic cells [1, 30]. RNF219 has been found to interact with the CCR4‐NOT complex and modulate its activity, thereby potentially influencing RNA metabolism [13, 14, 15]. Our study reveals that RNF219‐C31Y mutant condensates can sequester the CCR4‐NOT complex and reduce deadenylation activity in vitro. More evidence highlights that dysregulated mRNA clearance mechanisms have emerged as critical oncogenic drivers [31, 32]. We observed that the RNF219‐C31Y mutant upregulated the mRNA levels of genes including *FOS*, *JUN*, *EGR1*, *MYC* and *RHOB*, thereby promoting the proliferation of DLD‐1 cells. C31YΔCBD mutant, which retains LLPS capacity but loses CCR4‐NOT binding, functional assays in DLD‐1 cells revealed that this mutant failed to upregulate target mRNAs and showed no proliferation advantage. These results collectively demonstrate that C31Y promotes proliferation specifically through CCR4‐NOT‐dependent condensates (Figure 6).

Although our data link RING domain mutations to reduced CCR4‐NOT activity, the precise mechanism—whether structural disruption or condensate sequestration—requires further investigation. Future studies using ΔRING/CC1 variants under controlled phase separation conditions, or employing condensate‐modulating agents (e.g., crowding agents or dissolution drugs) [33, 34, 35], could dissect these contributions. Furthermore, although our focus was on RNF219‐CCR4‐NOT co‐condensation, the broader composition of these condensates (e.g., inclusion/exclusion of other RNA‐binding proteins or deadenylases) warrants exploration. Preliminary observations suggest that C31YΔCBD forms smaller, more numerous condensates compared with C31Y. Future proteomic analyses of purified condensates, alongside investigation of known RNF219 interactors (e.g., ORC1, SIRT1 and α‐catenin) [16, 17, 36], will clarify their roles in condensate biology.

## 4 Materials and Methods

### 4.1 Cell Culture

Human HeLa, HCT116, MHCC97H, A549, HEK293T and mouse MEF cells were cultured in Dulbecco's modified Eagle's (DMEM) medium (Sigma) supplemented with 10% fetal bovine serum (Ex‐Cell Bio) and 1% penicillin and streptomycin (Sangon Biotech). Human DLD‐1 cells were cultured in RPMI1640 medium (Hyclone) supplemented with 10% fetal bovine serum and 1% penicillin and streptomycin. All cells were cultured at 37°C in a 5% CO_2_ incubator.

### 4.2 Plasmids

For transient expression, pcDNA5/FRT‐TO expression vector (Invitrogen) was first modified to include either mClover3 or mCherry following the FLAG tag in frame. pcDNA5/FRT‐TO‐RNF219 and RNF219‐truncated plasmids were previously described [13]. FLAG‐tagged RNF219^Mut^ (C18S, C21S, V29F, C31Y, H35R, C38S, S39L, C41S, C52S, C55W, R88W, E109Q, P130S) and RNF219 constructs (ΔCC2 and C31YΔCC2) cDNAs were cloned into the pcDNA5/FRT‐TO vector. RNF219 mutants were generated using PCR mutagenesis. RNF219, RNF219^V29F^, RNF219^C31Y^ cDNAs were cloned into pcDNA5/FRT‐TO‐mClover3 or pcDNA5/FRT‐TO‐mCherry. RNF219, RNF219^V29F^, RNF219^C31Y^ and RNF219^C31Y^ΔCBD cDNAs were cloned into pSin‐FLAG. For expression and purification, RNF219 and its truncations (RING^WT^, RING^C31Y^, RING^WT^‐CC1, RING^C31Y^‐CC1 and CC1) were cloned into pET16b‐mCherry [37]. Light‐activated intracellular droplet condensation system [28] associated plasmids purchased by addgene (#122147, #122148). RNF219, and its mutants or truncations (RING^WT^, RING^C31Y^, RING^WT^‐CC1, RING^C31Y^‐CC1 and CC1) replaced the FUSN sequence in plasmid #122148. psPAX2 and pMD2.G have been previously described [38]. All plasmids used were verified by Sanger sequencing (Sangon Biotech).

### 4.3 Cell Transfection, Lentiviral Particle Preparation and Infection

Cells were transfected with PEI (Sigma) following the manufacturer's instructions with the following modifications. One million cells in 1 mL or 500 μL of growth media were plated in 1 well of a 6‐ or 12‐well plate, the PEI‐DNA mix was immediately added on top of the cells, and the media containing the transfection mix were replaced with fresh growth media 16 h after transfection. Cells were imaged 24 h after transfection.

Lentiviral particle preparation and infection were performed as described previously [38]. Briefly, around 75% confluent HEK293T cells in a 10 cm tissue culture plate were co‐transfected with 4 μg of light‐activated intracellular droplet condensation system associated plasmids of RNF219 and its mutants and truncations, or pSin‐FLAG‐RNF219 and its mutants, 3 μg of psPAX2 packaging plasmids, and 1 μg of pMD2.G envelope plasmids using HighGene (ABclonal). The media were replaced with fresh culture media after 16 h of transfection. The lentiviral supernatants were collected 48 and 72 h after transfection and filtered through the 0.45‐μm filters. HeLa cells were infected with the filtered lentiviral supernatants containing polybrene (8 μg/mL; Sigma). Cells were imaged 36 h after infection.

### 4.4 Antibodies

Rabbit polyclonal antibody against RNF219 was generated in‐house and described previously [13]; rabbit polyclonal antibody to CNOT1 was purchased from Proteintech (14276‐1‐AP); rabbit polyclonal antibody to CNOT7 was purchased from Abcam (ab195587); mouse monoclonal antibody to α‐TUBULIN was purchased from ABclonal (AC012); mouse monoclonal antibody to FLAG was purchased from HUABIO (M1403‐2).

### 4.5 Western Blot

Cell lysates were resolved in SDS–PAGE gels and transferred to polyvinylidene fluoride (PVDF) membrane. The membrane was then incubated with 1:50,000 anti‐α‐TUBULIN, 1:5000 anti‐RNF219, 1:3000 anti‐CNOT1, 1:6000 anti‐CNOT7 or 1:5000 anti‐FLAG antibody diluted in TBST and incubated overnight at 4°C. HRP‐conjugated secondary antibodies (RY15‐001, RY15‐002) were used at a dilution of 1:20,000. ECL substrate (Millipore) was applied to the membrane for imaging by autoradiography (Tanon).

### 4.6 Protein Stability and Half‐Life Assay

HEK293T cells were transfected with FLAG‐tagged RNF219 and mutants expressing. Following translation inhibition with 100‐μM CHX 24 h after transfection, cell lysates were collected at different time intervals, and protein expression levels were detected via WB. Greyscale values of FLAG and TUBULIN were analysed using ImageJ, and relative FLAG/TUBULIN ratios were used to plot protein degradation curves. The protein half‐life was defined as the time required for levels to decrease to 50% of the initial value.

### 4.7 Immunofluorescence

HeLa cells were chosen for immunofluorescence and live‐cell imaging due to their large nuclear morphology and robust adhesion to coverslips. Cells grown on coverslips were fixed with 4% paraformaldehyde in PBS for 10 min at room temperature. After three washes in PBS for 5 min, cells were permeabilized with 0.2% Triton X‐100 in PBS for 5 min at room temperature. Following rinses with PBS, cells were blocked in blocking buffer (2% bovine serum albumin and 0.3% Triton X‐100) for at least 1 h at room temperature and incubated with diluted primary antibodies (1:200 anti‐CNOT1, 1:800 anti‐CNOT7 and 1:1000 anti‐FLAG) in blocking buffer overnight at 4°C. After three washes in PBS, coverslips were incubated at room temperature with secondary antibodies (1:2000 goat anti‐mouse IgG Alexa Fluor 488, and 1:2000 goat anti‐mouse IgG Alexa Fluor 555) in the dark for 1 h, followed by three washes with PBS. Coverslips were mounted on slides using the VECTASHIELD Mounting Medium with DAPI (4′,6‐diamidino‐2‐phenylindole). Three‐dimensional images were acquired using a Zeiss LSM 700 confocal microscope with 63×, 1.4 numerical aperture (NA) oil immersion objective lens using Zen Light Edition acquisition software and charge‐coupled device (CCD) camera. Images were postprocessed using Zen Light Edition.

### 4.8 Live‐Cell Imaging

Cells were grown in a 12‐well plate and imaged using the AxioCam MRm detector on Axio Observer Z1 (Zeiss) in an incubation chamber to maintain culture conditions (37°C, 5% CO_2_). ZEN black edition version 2.3 (Zeiss) was used for acquisition. Images were acquired with the AxioCam MRm camera with a 20× objective. Raw images were processed using ZEN 2.3 (Zeiss).

### 4.9 Recombinant Protein Purification

Recombinant plasmids were transformed into the *E. coli* BL‐21 expression system. Mid‐log phase of the transformed BL‐21 cells was induced by 1‐mM isopropyl‐β‐D‐thiogalactopyranoside (IPTG) for 16 h at 18°C to express the proteins of interest. To purify the recombinant proteins, cell pellets from 500 mL of culture were resuspended in 40 mL of lysis buffer (50‐mM NaH_2_PO_4_ [pH 8.0], 300‐mM NaCl, 10‐mM imidazole and 0.05% Tween 20) in the presence of the protease inhibitor phenyl methane sulfonyl fluoride (PMSF) and homogenised using a high‐pressure homogeniser for five cycles at 10,000 mPa. The crude lysate was cleared by centrifugation at 20,000 g for 1 h at 4°C. 0.5 mL Ni‐nitrilotriacetic acid (NTA) agarose (smart‐lifesciences, SA101005) preequilibrated with lysis buffer was then added to the cleared lysate. After overnight incubation at 4°C, the lysate agarose slurry was washed five times with 10 mL of wash buffer (50‐mM NaH_2_PO_4_ [pH 8.0], 300‐mM NaCl, 30‐mM imidazole and 0.05% Tween 20). Protein was eluted with 5 mL of elution buffer (50‐mM NaH_2_PO_4_ [pH 8.0], 300‐mM NaCl, 250‐mM imidazole and 0.05% Tween 20). The purified recombinant proteins were analysed by Coomassie‐stained SDS– polyacrylamide gel electrophoresis (PAGE) and dialyzed against droplet formation buffer (50‐mM tris–HCl [pH 7.5], 10% glycerol and 1‐mM dithiothreitol).

### 4.10 In Vitro Droplet Assay

In vitro droplet assay was performed as previously described [37]. Briefly, recombinant proteins were concentrated and desalted using Amicon Ultra centrifugal filters (Millipore). Recombinant proteins (10 μM) were then added to droplet formation buffer containing indicated concentration of salt in 10% PEG‐8000 (Sigma). The protein solution was immediately loaded onto a coverslip and imaged with a Zeiss microscope with a 20× objective.

### 4.11 Immunoprecipitation

Immunoprecipitation was performed as previously described [38]. Briefly, cells were lysed in 420‐mM NaCl containing lysis buffer (20‐mM HEPES, pH 7.4, 25% glycerol, 420‐mM NaCl, 1.5‐mM MgCl_2_, 0.2‐mM EDTA, 1‐mM DTT) supplemented with protease inhibitor cocktail (Sigma) at 4°C. After centrifugation, the balance buffer (20‐mM HEPES, pH 7.4, 1‐mM MgCl_2_ and 10‐mM KCl) was added to the supernatant to make the final NaCl concentration 300 mM. One percent of total lysate was saved as input. Equal amounts of total protein were used for IP using anti‐FLAG M2 magnetic beads (Sigma–Aldrich) for 4 h at 4°C. The beads were spun down and washed three times with the wash buffer (10‐mM HEPES, pH 7.4, 1.5‐mM MgCl_2_, 150‐mM NaCl, 10‐mM KCl and 0.02% Triton X‐100) before boiling in the 2× SDS loading buffer.

### 4.12 In Vitro Deadenylation Assay

HEK293T were transfected with pcDNA5/FRT‐TO‐RNF219^WT^ and pcDNA5/FRT‐TO‐RNF219^C31Y^ expressing FLAG‐RNF219 and were subjected to FLAG purification. The FLAG‐M2 beads‐bound bait protein, and its interacting factors were incubated with 200 nM FAM labelled synthetic RNA substrate 20mer‐A20 (Sangon Biotech) in reaction buffer [20‐mM PIPES pH 6.8, 10‐mM KCl, 40‐mM NaCl and 2‐mM Mg(OAc)_2_] at 37°C. A rotator was used to ensure complete reaction, operating at a speed of 60 rpm. Samples were collected at different time points and analysed on a 20% polyacrylamide (20% [w/v] 19:1 acrylamide‐bis acrylamide) denaturing gel with 7‐M urea. The images were analysed with a fluorescence imager (Tanon).

### 4.13 Real‐Time Quantitative PCR (RT‐qPCR)

Total RNA was isolated with RNA isolater Total RNA Extraction Reagent (Vazyme, R401‐01). cDNAs were synthesised with the *EasyScript* One‐Step gDNA Removal and cDNA Synthesis SuperMix (Transgen, AE311‐02). The expression levels were measured with 2× Universal SYBR Green Fast qPCR Mix (ABclonal, RK21203) on CFX96 (Bio‐Rad). The relative expression levels of genes of interest were normalised to the expression of the housekeeping gene *GAPDH*. Relative fold changes in gene expression were calculated using the ΔΔCT (threshold cycle) method. The information on the primers used in this study was provided in Table S2.

### 4.14 Quantifying Viable Cell Numbers in Proliferation

Cells were seeded in 96‐well plates at 1000 cells/well. Ten microlitres of CCK‐8 (Yeasen, 40203ES76) was added to each well at time points of 0, 24, 48, 72 and 96 h of cell growth. Then, after 2 h of incubation at 37°C and 5% CO_2_, the OD 450 values were measured using an imaging reader (Allsheng, FlexA‐200).

### 4.15 Clonogenic Assay

The clonogenic assay was performed by seeding 200 cells/well into 12‐well plates with the indicated treatments. Fourteen days after seeding, colonies were fixed by 4% formaldehyde and stained with 0.1% crystal violet dye (Sangon Biotech) dissolved in 2% ethanol.

### 4.16 Clinical Data Analysis

The analyses of *RNF219* alteration event frequency genes were performed using the Genomic Data Commons Data Portal (https://portal.gdc.cancer.gov/). The analyses of *RNF219* mutation were performed using the cBioPortal database (http://www.cbioportal.org/).

## Author Contributions

C.L. and Z.L. designed the research and supervised the project. C.C. performed the experiments and analysed the data. C.G. and K.F. revised and discussed the paper. C.L. and Z.L. provided resources. C.C. and Z.L. wrote the paper.

## Ethics Statement

The authors have nothing to report.

## Conflicts of Interest

The authors declare no conflicts of interest.

## Supporting information


**Figure S1.** *RNF219* mutation frequency and protein species conservation. (A) Schematic representation of the RNF219‐CCR4‐NOT complex. (B) Statistical analysis of *RNF219* mutations from The Cancer Genome Atlas (TCGA). (C) Analysis of RNF219 sequence conservation across vertebrates. Mark the completely identical parts in yellow and mark the parts with a similarity greater than 50% in blue. Protein sequences were aligned using Vector NTI.

**Figure S2.** RNF219^C31Y^ stability and condensate formation in cells. (A) Western blot showing the protein levels of endogenous or transfected FLAG‐tagged RNF219 in HEK293T cells or HeLa cells. Anti‐RNF219 and anti‐FLAG antibodies were used to detect RNF219 protein levels. TUBULIN was used as a loading control. *, non‐specific band. (B) Western blot showing the protein levels of transfected FLAG‐tagged RNF219 and mutants in HEK293T treated with 100‐μM cycloheximide (CHX) for indicated time. FLAG‐tagged RNF219 mutant proteins detected via anti‐FLAG antibody, TUBULIN was used as a loading control. The relative intensity of FLAG/TUBULIN was qualified using ImageJ. (C) Live‐cell imaging of HeLa cells expressing FLAG‐tagged RNF219^WT^‐mClover3, RNF219^V29F^‐mClover3 and RNF219^C31Y^‐mClover3. Scale bar, 10 μm. (D) Quantification result of (C) was shown. Green was solution, yellow was plaque, and blue was puncta (*n* ≥ 50). (E) Live‐cell imaging of HeLa cells expressing FLAG‐tagged RNF219^WT^‐mCherry and RNF219^V29F^‐mCherry and RNF219^C31Y^‐mCherry. Scale bar, 10 μm. (F) Quantification result of (E) was shown. Green was solution, yellow was plaque, and blue was puncta (*n* ≥ 50). (G) Live‐cell imaging of HEK293T, DLD‐1, HCT116, A549, MHCC97H and MEF expressing RNF219^C31Y^‐mClover3. Scale bar, 10 μm. (H) Quantification result of (G) was shown, blue was puncta (*n* ≥ 50).

**Figure S3.** Expression of RNF219 constructs and mCherry fusion recombinant proteins. (A) Western blot showing the protein levels of transfected FLAG‐tagged RNF219 full length (FL) and truncated proteins, ΔRING, Δ(RING‐CC1), Δ(RING‐CC1‐CC2) in HeLa cells. FLAG‐tagged RNF219 constructs detected via anti‐FLAG antibody. *, non‐specific band. (B) Western blot showing the protein levels of transfected FLAG‐tagged RNF219 FL and constructs (ΔCC2 and C31YΔCC2) in HeLa cells. FLAG‐tagged RNF219 constructs detected via anti‐FLAG antibody. (C) SDS‐PAGE results of purified recombinant RING^WT^‐mCherry, RING^C31Y^‐mCherry, RING^WT^‐CC1‐mCherry, RING^C31Y^‐CC1‐mCherry, CC1‐mCherry and mCherry protein. Arrows indicate recombinant protein.

**Figure S4.** The verification of FLAG purification and cell lines. (A) Levels of FLAG‐tagged RNF219^WT/C31Y^, CNOT1 and CNOT7 in the purified immuneprecipitates were examined by Western blotting. (B) Western blot showing the protein levels of FLAG‐tagged RNF219 and mutants in DLD‐1 cells. FLAG‐tagged RNF219 mutant proteins detected via anti‐FLAG antibody, TUBULIN was used as a loading control. (C) Representative confocal images of DLD‐1 cells. Immunostained with anti‐FLAG and anti‐CNOT7 antibodies, DNA was counterstained using DAPI. Scale bar, 5 μm.

**Table S1.** Recombinant DNA used in this study.
**Table S2.** Primers used in this study.

## Data Availability

The data that support the findings of this study are available on request from the corresponding author. The data are not publicly available due to privacy or ethical restrictions.
