# Supplementary figures and images for: RNF219 RING Finger Domain Mutants Drive Phase Separation to Encapsulate CCR4‐NOT and Promote Cell Proliferation
Source: Cell Prolif. 2025 Jun 11;59(1):e70072. doi: 10.1111/cpr.70072 (PMC12774619; doi:10.1111/cpr.70072)

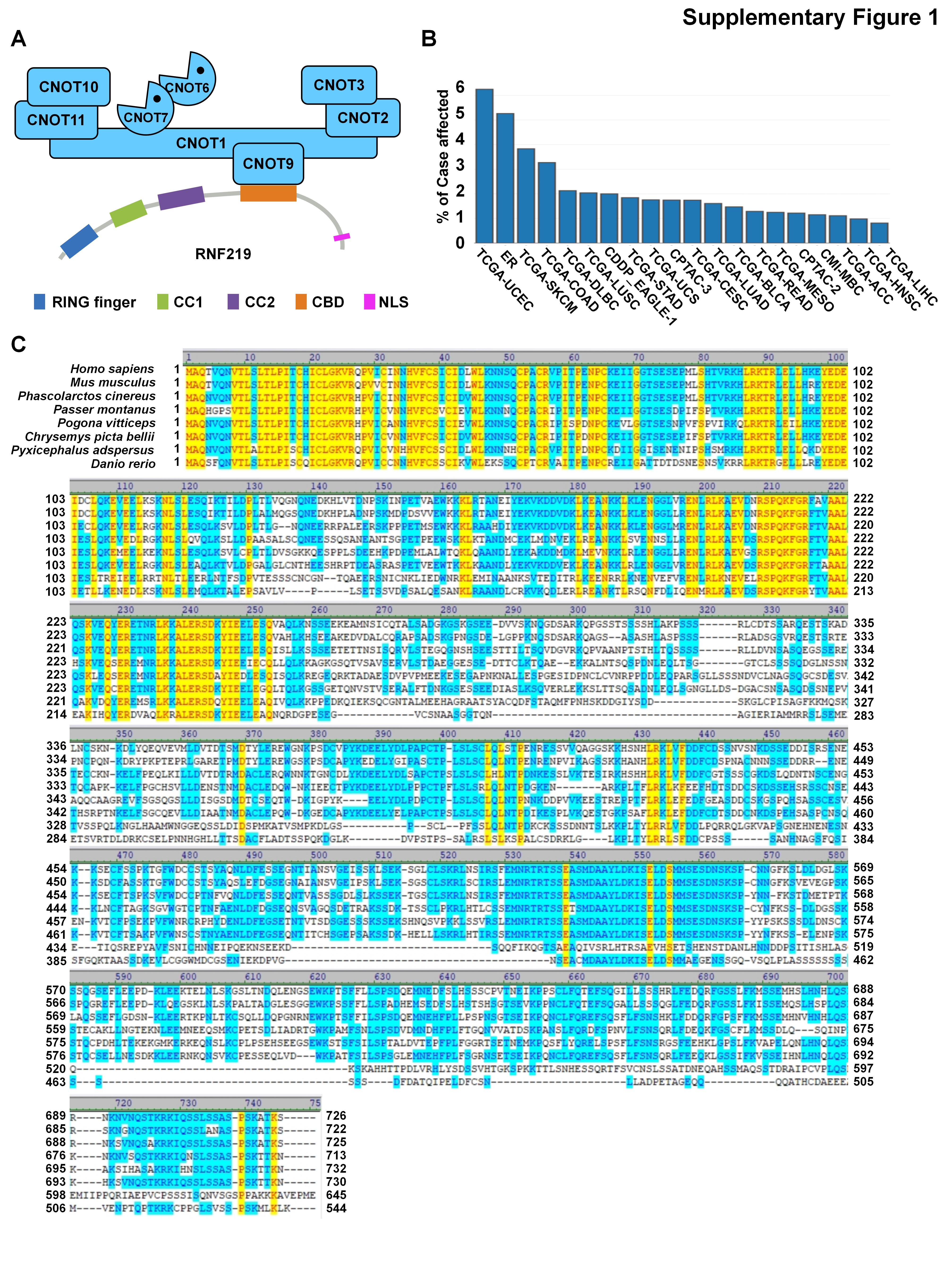

Supplement: Supplementary file 1 — Figure S1. RNF219 mutation frequency and protein species conservation. (A) Schematic representation of the RNF219‐CCR4‐NOT complex. (B) Statistical analysis of RNF219 mutations from The Cancer Genome Atlas (TCGA). (C) Analysis of RNF219 sequence conservation across vertebrates. Mark the completely identical parts in yellow and mark the parts with a similarity greater than 50% in blue. Protein sequences were aligned using Vector NTI. [file CPR-59-e70072-s001.jpg]

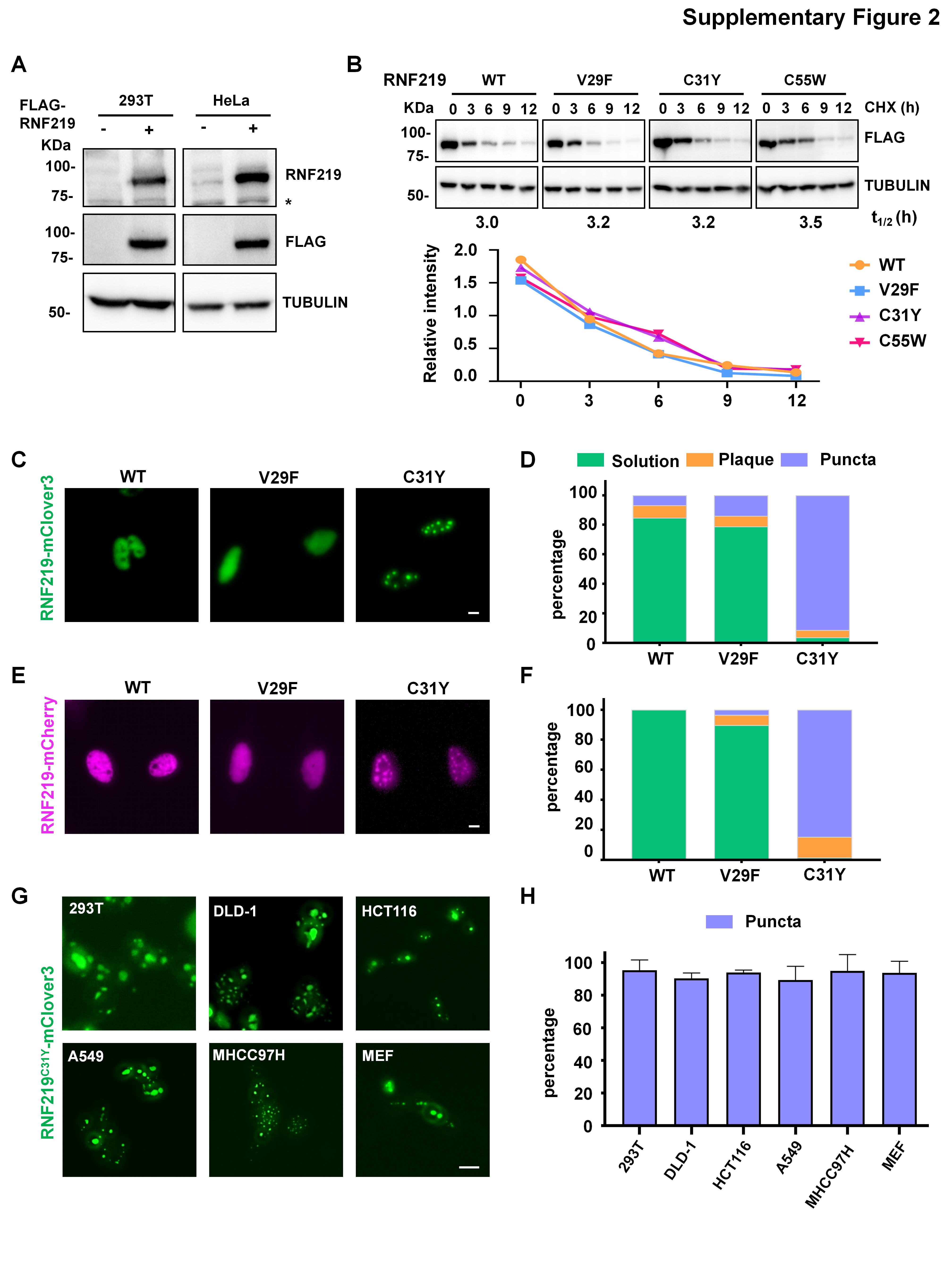

Supplement: Supplementary file 2 — Figure S2. RNF219C31Y stability and condensate formation in cells. (A) Western blot showing the protein levels of endogenous or transfected FLAG‐tagged RNF219 in HEK293T cells or HeLa cells. Anti‐RNF219 and anti‐FLAG antibodies were used to detect RNF219 protein levels. TUBULIN was used as a loading control. *, non‐specific band. (B) Western blot showing the protein levels of transfected FLAG‐tagged RNF219 and mutants in HEK293T treated with 100‐μM cycloheximide (CHX) for indicated time. FLAG‐tagged RNF219 mutant proteins detected via anti‐FLAG antibody, TUBULIN was used as a loading control. The relative intensity of FLAG/TUBULIN was qualified using ImageJ. (C) Live‐cell imaging of HeLa cells expressing FLAG‐tagged RNF219WT‐mClover3, RNF219V29F‐mClover3 and RNF219C31Y‐mClover3. Scale bar, 10 μm. (D) Quantification result of (C) was shown. Green was solution, yellow was plaque, and blue was puncta (n ≥ 50). (E) Live‐cell imaging of HeLa cells expressing FLAG‐tagged RNF219WT‐mCherry and RNF219V29F‐mCherry and RNF219C31Y‐mCherry. Scale bar, 10 μm. (F) Quantification result of (E) was shown. Green was solution, yellow was plaque, and blue was puncta (n ≥ 50). (G) Live‐cell imaging of HEK293T, DLD‐1, HCT116, A549, MHCC97H and MEF expressing RNF219C31Y‐mClover3. Scale bar, 10 μm. (H) Quantification result of (G) was shown, blue was puncta (n ≥ 50). [file CPR-59-e70072-s002.jpg]

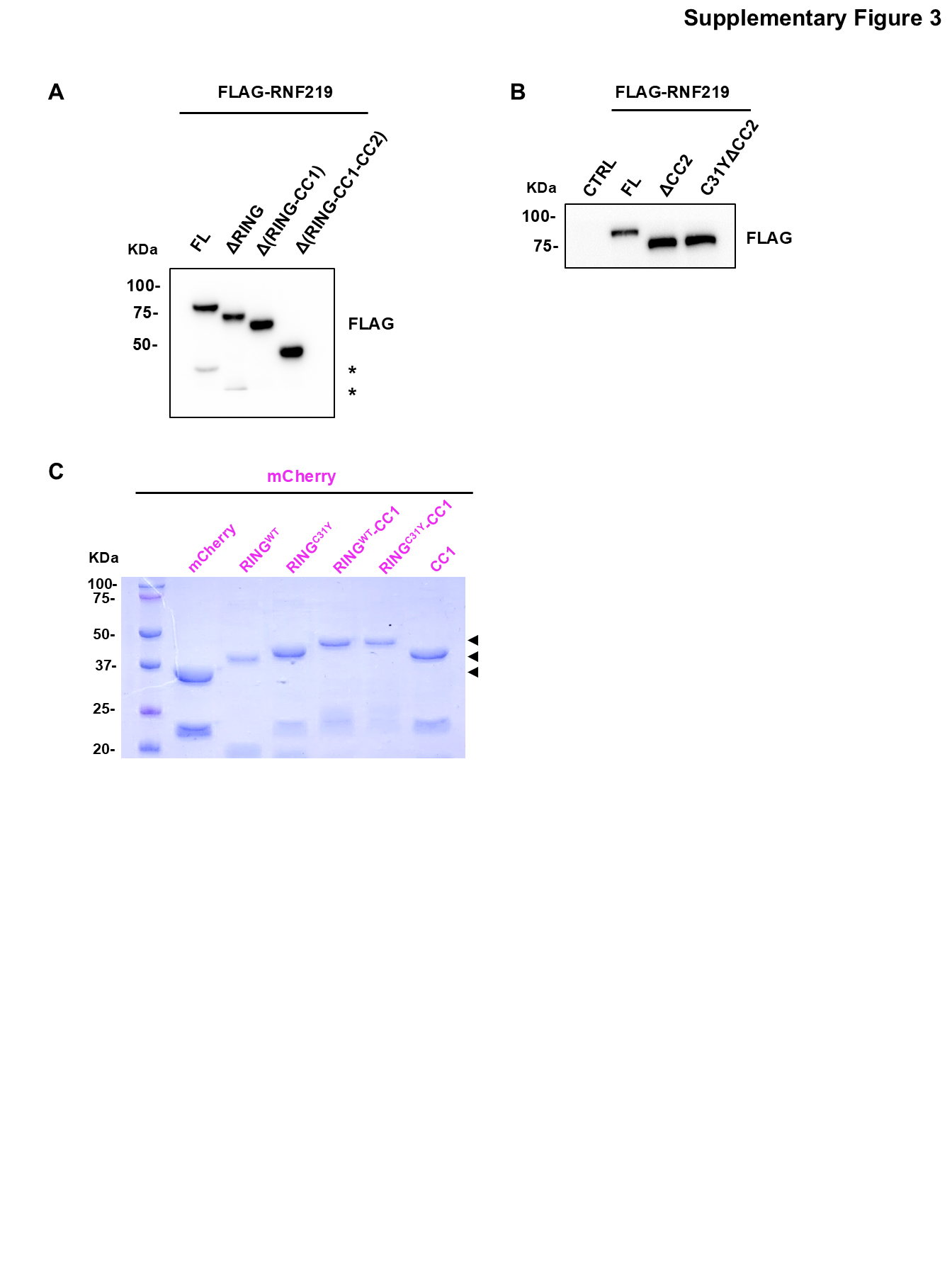

Supplement: Supplementary file 3 — Figure S3. Expression of RNF219 constructs and mCherry fusion recombinant proteins. (A) Western blot showing the protein levels of transfected FLAG‐tagged RNF219 full length (FL) and truncated proteins, ΔRING, Δ(RING‐CC1), Δ(RING‐CC1‐CC2) in HeLa cells. FLAG‐tagged RNF219 constructs detected via anti‐FLAG antibody. *, non‐specific band. (B) Western blot showing the protein levels of transfected FLAG‐tagged RNF219 FL and constructs (ΔCC2 and C31YΔCC2) in HeLa cells. FLAG‐tagged RNF219 constructs detected via anti‐FLAG antibody. (C) SDS‐PAGE results of purified recombinant RINGWT‐mCherry, RINGC31Y‐mCherry, RINGWT‐CC1‐mCherry, RINGC31Y‐CC1‐mCherry, CC1‐mCherry and mCherry protein. Arrows indicate recombinant protein. [file CPR-59-e70072-s004.tif]

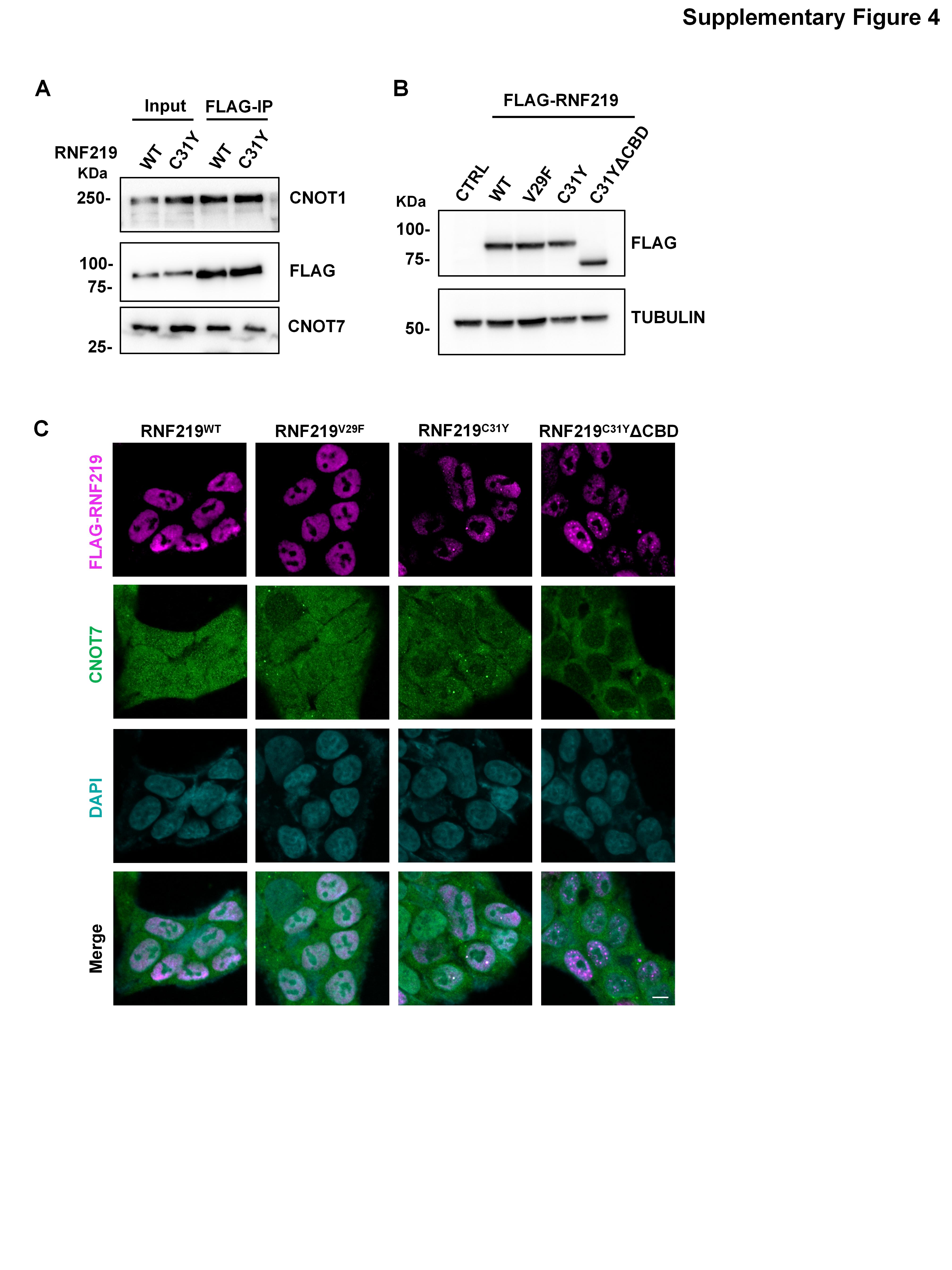

Supplement: Supplementary file 4 — Figure S4. The verification of FLAG purification and cell lines. (A) Levels of FLAG‐tagged RNF219WT/C31Y, CNOT1 and CNOT7 in the purified immuneprecipitates were examined by Western blotting. (B) Western blot showing the protein levels of FLAG‐tagged RNF219 and mutants in DLD‐1 cells. FLAG‐tagged RNF219 mutant proteins detected via anti‐FLAG antibody, TUBULIN was used as a loading control. (C) Representative confocal images of DLD‐1 cells. Immunostained with anti‐FLAG and anti‐CNOT7 antibodies, DNA was counterstained using DAPI. Scale bar, 5 μm. [file CPR-59-e70072-s003.jpg]
