# Supplementary material for: RNF219 RING Finger Domain Mutants Drive Phase Separation to Encapsulate CCR4‐NOT and Promote Cell Proliferation
Source: Cell Prolif. 2025 Jun 11;59(1):e70072. doi: 10.1111/cpr.70072 (PMC12774619; doi:10.1111/cpr.70072)
Supplement: Supplementary file 5 — Table S1. Recombinant DNA used in this study. Table S2. Primers used in this study. [file CPR-59-e70072-s005.doc]

**Supplementary Files**

**Supplementary Table 1. Recombinant DNA used in this study**

| Plasmids | Source |
| --- | --- |
| pcDNA5/FRT-TO | Invitrogen |
| pcDNA5/FRT-TO-RNF219^WT^ | Du et al. 2020 |
| pcDNA5/FRT-TO-RNF219^C18S^ | This paper |
| pcDNA5/FRT-TO-RNF219^C21S^ | This paper |
| pcDNA5/FRT-TO-RNF219^V29F^ | This paper |
| pcDNA5/FRT-TO-RNF219^C31Y^ | This paper |
| pcDNA5/FRT-TO-RNF219^H35R^ | This paper |
| pcDNA5/FRT-TO-RNF219^C38S^ | This paper |
| pcDNA5/FRT-TO-RNF219^S39L^ | This paper |
| pcDNA5/FRT-TO-RNF219^C41S^ | This paper |
| pcDNA5/FRT-TO- RNF219^C52S^ | This paper |
| pcDNA5/FRT-TO-RNF219^C55W^ | This paper |
| pcDNA5/FRT-TO-RNF219^R88W^ | This paper |
| pcDNA5/FRT-TO-RNF219^E109Q^ | This paper |
| pcDNA5/FRT-TO-RNF219^P130S^ | This paper |
| pcDNA5/FRT-TO-RNF219-ΔRING | Du et al. 2020 |
| pcDNA5/FRT-TO-RNF219-Δ(RING-CC1) | Du et al. 2020 |
| pcDNA5/FRT-TO-RNF219-Δ(RING-CC1-CC2) | Du et al. 2020 |
| pcDNA5/FRT-TO-RNF219-ΔCC2 | This paper |
| pcDNA5/FRT-TO-RNF219^C31Y^-ΔCC2 | This paper |
| pcDNA5/FRT-TO-RNF219^C31Y^-ΔCBD | This paper |
| pcDNA5/FRT-TO-RNF219^WT^-mClover3 | This paper |
| pcDNA5/FRT-TO-RNF219^V29F^-mClover3 | This paper |
| pcDNA5/FRT-TO-RNF219^C31Y^-mClover3 | This paper |
| pcDNA5/FRT-TO-RNF219^WT^-mCherry | This paper |
| pcDNA5/FRT-TO-RNF219^V29F^-mCherry | This paper |
| pcDNA5/FRT-TO-RNF219^C31Y^-mCherry | This paper |
| pET16b-mCherry | Guo et al., 2020 |
| pET16b-RNF219-RING^WT^-mCherry | This paper |
| pET16b-RNF219-RING^C31Y^-mCherry | This paper |
| pET16b-RNF219-RING^WT^-CC1-mCherry | This paper |
| pET16b-RNF219-RING^C31Y^-CC1-mCherry | This paper |
| pET16b-RNF219-CC1-mCherry | This paper |
| pHR-SFFVp-RNF219-RING^WT^-mCherry-SspB | This paper |
| pHR-SFFVp-RNF219-RING^C31Y^-mCherry-SspB | This paper |
| pHR-SFFVp-RNF219-RING^WT^-CC1-mCherry-SspB | This paper |
| pHR-SFFVp-RNF219-RING^C31Y^-CC1-mCherry-SspB | This paper |
| pHR-SFFVp-RNF219-CC1-mCherry-SspB | This paper |
| pHR-SFFVp-NLS-iLID-EGFP-FTH1 | Bracha et al., 2018 |
| pSin-FLAG | This paper |
| pSin-FLAG-RNF219^WT^ | This paper |
| pSin-FLAG-RNF219^V29F^ | This paper |
| pSin-FLAG-RNF219^C31Y^ | This paper |
| pSin-FLAG-RNF219^C31Y^-ΔCBD | This paper |
| psPAX2 | Lin et al., 2010 |
| pMD2.G | Lin et al., 2010 |

**Supplementary Table 2. Primers used in this study**

| Primer | Sequence |
| --- | --- |
| FOS-F | AGGAGGGAGCTGACTGATACACTC |
| FOS-R | GCAGACTTCTCATCTTCTAGTTGGTC |
| JUN-F | TGACTGCAAAGATGGAAACG |
| JUN-R | CAGGGTCATGCTCTGTTTCA |
| EGR1-F | TACGAGCACCTGACCGCA |
| EGR1-R | CACCAGCACCTTCTCGTTGTT |
| MYC-F | CCACAGCAAACCTCCTCACAG |
| MYC-R | GCAGGATAGTCCTTCCGAGTG |
| RHOB-F | CTGCTGCAAGGTGCTATGAG |
| RHOB-R | ACACGGGTCTCCCCTTCT |
| GAPDH-F | TGCACCACCAACTGCTTAGC |
| GAPDH-R | GAGGGGCCATCCACAGTCTTC |
